# Intracellular Delivery of Active Proteins by Polyphosphazene Polymers

**DOI:** 10.3390/pharmaceutics13020249

**Published:** 2021-02-10

**Authors:** Bareera Qamar, Melani Solomon, Alexander Marin, Thomas R. Fuerst, Alexander K. Andrianov, Silvia Muro

**Affiliations:** 1 College of Mathematical and Natural Sciences, University of Maryland, College Park, MD 20742, USA; bqamar@terpmail.umd.edu; 2 Institute for Bioscience and Biotechnology Research, University of Maryland, College Park, MD 20742, USA; msolom4@umd.edu (M.S.); amarin1@umd.edu (A.M.); tfuerst@umd.edu (T.R.F.); 3 Institute of Catalonia for Research and Advanced Studies, 08010 Barcelona, Spain; 4 Institute for Bioengineering of Catalonia of the Barcelona Institute of Science and Technology, 08028 Barcelona, Spain

**Keywords:** polyphosphazene polymers, intracellular protein delivery, endosomal escape, cytosolic delivery, intracellular delivery of antibody, delivery of apoptotic peptides, cytotoxicity

## Abstract

Achieving intracellular delivery of protein therapeutics within cells remains a significant challenge. Although custom formulations are available for some protein therapeutics, the development of non-toxic delivery systems that can incorporate a variety of active protein cargo and maintain their stability, is a topic of great relevance. This study utilized ionic polyphosphazenes (PZ) that can assemble into supramolecular complexes through non-covalent interactions with different types of protein cargo. We tested a PEGylated graft copolymer (PZ-PEG) and a pyrrolidone containing linear derivative (PZ-PYR) for their ability to intracellularly deliver FITC-avidin, a model protein. In endothelial cells, PZ-PYR/protein exhibited both faster internalization and higher uptake levels than PZ-PEG/protein, while in cancer cells both polymers achieved similar uptake levels over time, although the internalization rate was slower for PZ-PYR/protein. Uptake was mediated by endocytosis through multiple mechanisms, PZ-PEG/avidin colocalized more profusely with endo-lysosomes, and PZ-PYR/avidin achieved greater cytosolic delivery. Consequently, a PZ-PYR-delivered anti-F-actin antibody was able to bind to cytosolic actin filaments without needing cell permeabilization. Similarly, a cell-impermeable Bax-BH3 peptide known to induce apoptosis, decreased cell viability when complexed with PZ-PYR, demonstrating endo-lysosomal escape. These biodegradable PZs were non-toxic to cells and represent a promising platform for drug delivery of protein therapeutics.

## 1. Introduction

Delivery of therapeutic compounds to the sub-cellular compartments where their targets reside is essential towards achieving maximal therapeutic efficacy [1]. This is especially relevant for a majority of biological therapeutics that are aimed to elicit their action through interactions with molecular targets within cells. Among these, protein therapeutics are being widely developed due to the seminal roles proteins play as modulators of numerous physiological processes in the body [2,3]. However, while significant progress has been made towards the development of protein therapeutics for applications requiring interactions on cell surfaces or with secreted molecules (e.g., antibodies), some key challenges to the intracellular delivery of protein therapeutics still remain today [4,5].

A major hurdle to the delivery of proteins and other macromolecules inside cells is their large size and hydrophilicity that prevents their diffusion across lipid cell membranes [3,5]. While genetic approaches that rely on the expression of therapeutic antibodies within cells (e.g., intrabodies, nanobodies) can potentially overcome this limitation, they are met with challenges of their own such as integration into the genome, carcinogenicity, immunogenicity, etc. [5,6]. Alternatively, proteins can be conjugated with affinity molecules that can interact with specific cell receptors on the cell surface, leading to their internalization in endocytic vesicles [3]. However, once they enter cells, they most often stay trapped in these vesicles, which leads to their lysosomal degradation [5]. Endo-osmolytic agents have been incorporated in delivery systems to enable endosomal disruption and cargo release, yet the efficiency of such processes remains low and some of them are toxic to cells [3,5,7]. Other approaches use non-endocytic mechanisms to deliver cargo to cells such as through conjugation with cell penetrating peptides (CPPs), also termed protein transduction domains (PTDs); however, these mostly suffer from toxicity, unclear mechanistic understanding, and low efficiency [3,5,7,8,9]. Recent developments in the field have led to the design of less toxic and more stable cyclic CPPs, CPP prodrugs that can be programmed to release their cargo at specific tissue sites, as well as fusions with subcellular targeting moieties to achieve organelle targeting or with fusogenic peptides to achieve endosomal escape [10]. In fact, CPPs are under testing in several clinical trials where the cargo is conjugated to the CPP or generated as a chimeric fusion [10]. However, sparing some exceptions [11,12,13], such strategies involve covalent attachment of targeting/delivery moieties to the protein cargo or development of chimeras, which can affect the protein folding, sites of interaction with substrates or partner proteins, and ultimately cargo activity, requiring synthesis and optimization on a case-by-case basis [3,5]. Additional strategies utilizing click chemistry have gained increasing attention due to their relative tunability based on the addition of modifiable units to the protein structure, which can then be conjugated to targeting polymers [14]. However, while they can achieve intracellular targeting of cargo, similar concerns apply and these techniques do not offer a platform technology for protein delivery since each particular protein cargo requires their own residue-specific engineering/conjugating scheme. Some polymer-based systems for protein delivery use cationic polymer scaffolds that were traditionally used for gene delivery, with functionalization of groups such as boronate or guanidium on the polymer to increase attachment of protein cargo [2]. However, their back-bones still raise toxicity concerns.

Utilization of other carriers systems as delivery vehicles may overcome these challenges [15]. Fusogenic liposomes [16], inorganic nanoparticles [17], polymer-based nanoparticles [18], solid lipid nanoparticles [19], nucleic acids-based particles [20,21], and exosomes [22] are examples of carrier systems that have been used to deliver a variety of protein therapeutics such as enzymes to lysosomes [18,23], transcription factors to the nucleus [21], ribonucleoprotein complexes [19], and protein therapeutics to the cytosol [17,19,20]. Yet, these approaches face limitations such as loss of protein activity or stability during formulation, charge or size restrictions for certain cargo types, therapeutic loading limitations, toxicity of the carrier, inability to escape endosomes for several nanocarriers, or rapid clearance from the circulation [2,5,18].

Hence, while a number of strategies are available to achieve intracellular delivery of proteins, the development of a non-covalent approach that can provide sufficient therapeutic load without affecting protein structure or activity, can be adapted to a variety of cargo and cell types, and can avoid cytotoxicity are limited [11,12,13,24,25,26], warranting the development of other delivery systems. Water-soluble polyphosphazenes (PZs) are hybrid organic-inorganic polyelectrolyte-type polymers that are attaining increasing recognition as multifunctional drug delivery vehicles [27,28,29]. Their unique macromolecular structure consists of an alternating phosphorus and nitrogen backbone and organic side groups, which provides for hydrolytic degradability under physiological conditions [30] and enables self-assembly with macromolecular drugs, including proteins, and some biological targets through non-covalent interactions [27,31,32,33,34]. PZ polymers have been formulated mostly as vaccine adjuvants [35] or in supramolecular assemblies including micelles, nanoparticles, hydrogels etc., hence they are flexible systems with multiple applications accorded by adjusting the side groups for different applications [36].

We have reported the synthesis of two families of water-soluble PZs, which combine carboxylic acid functionalities with hydrophilic moieties, such as pyrrolidone (PZ-PYR) [31,37] and graft poly(ethylene glycol) (PEG) chains (PZ-PEG) [32,33]. One of the most important design features of these polymers is their excellent solubility in aqueous solutions at near physiological pH [31,32]. PZ polymers are miscible with water in a broad range of ratios and form clear, low viscosity solutions at concentrations ranging from 0.1 to 2 mg/mL, at which they have been used as injectable formulations [27,35]. We have shown these hydrolytically degradable carriers facilitate uptake of protein cargo by cells in culture, in particular, avidin used as a model protein [31,33]. However, their relative efficiency to deliver protein cargo to cells has not been compared, their behavior has not been explored comparatively in different cells types, e.g., control vs. diseased cells, and the mechanism for their uptake within cells has not been reported. Most importantly, their intracellular trafficking after entering cells remains largely unknown. Due to the presence of carboxylic groups in the polymer that could disrupt endosome membranes due to protonation at acidic pH conditions of the endosome [38], it is possible that PZs could release endocytosed cargo to the cytosol. Yet, PZs have never been examined in this context.

The results reported here demonstrate the differential behavior of each of these PZ polymers depending on the cell type investigated, their versatility regarding the uptake mechanisms they use to enter cells, and their ability to deliver active protein cargo to the cytosol, while being non-toxic.

## 2. Materials and Methods

*Reagents*. Cells used in this study were human oral adenosquamous carcinoma Cal27 cells from American Type Culture Collection (Manassas, VA, USA) and Human Umbilical Vein Endothelial Cells (HUVECs) from Lonza Walkersville, Inc (Walkersville, MD, USA). FITC-avidin lyophilized powder from egg white was from Sigma-Aldrich (St. Louis, MO, USA), biotinylated mouse IgG was from BD Biosciences (PharminGen, San Jose, CA, USA), Texas-Red dextran (10 kDa) was from Molecular Probes (Carlsbad, CA, USA), anti-Early Endosome Antigen-1 (EEA-1) antibody was from Sigma-Aldrich (St. Louis, MO, USA), Texas-red conjugated secondary antibodies were from Life Technologies (Carlsbad, CA, USA), anti-F-actin antibody was from Bioss antibodes (Woburn, MA, USA), Bax-BH3 peptide (STKKLSECLKRIGDELDSNM) was from AnaSpec Inc. (Fremont, CA, USA), phosphate buffered saline, pH 7.4 (PBS), and 4′, 6-diamidino-2-phenylindole (DAPI) from Invitrogen (Carlsbad, CA, USA). LIVE/DEAD Mammalian Kit was from molecular Probes (Eugene, OR, USA). Medium M199 was from Invitrogen (Carlsbad, CA, USA) and Dulbecco’s Modified Eagle’s Medium from GibcoBRL (Grand Island, NY, USA).

*Cell Culture.* HUVECs were cultured in M199 supplemented with 15% fetal bovine serum, 2 mM l-glutamine, 15 µg/mL endothelial cell growth supplement, 100 µg/mL heparin, 100 U/mL penicillin, and 100 µg/mL streptomycin. Cal27 cells were cultured in DMEM medium supplemented with 10% fetal bovine serum and 1% penicillin/streptomycin. Both cell types were seeded on gelatin-coated glass coverslips and grown to confluence at 37 °C, 5% CO_2_, and 95% relative humidity.

**Figure 1:**
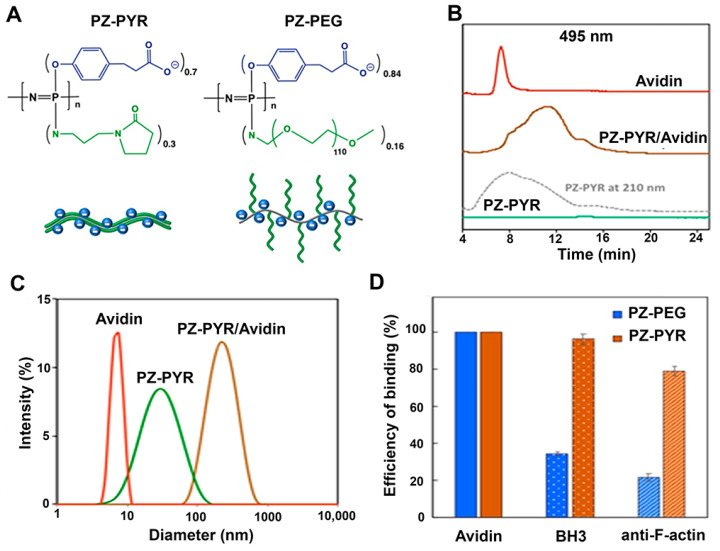
Schematics and characterization of polyphosphazenes (PZ)/protein complexes. (**A**) Chemical structures of PZ-PYR and PZ-PEG polymers and their schematic presentations. (**B**) Representative AF4 profiles of FITC-avidin, PZ-PYR, and PZ-PYR/FITC-avidin as detected at 495 nm (PZ-PYR profile at 210 nm detection is shown for comparison purposes). (**C**) Dynamic light scattering profiles of FITC-avidin, PZ-PYR, and PZ-PYR/FITC-avidin (25 mM phosphate buffer, pH 7.4). (**D**) Efficiency of protein or peptide binding to PZ-PEG and PZ-PYR expressed as a percent of bound molecules of their total amount for FITC-avidin, Bax-BH3 peptide, and anti-F-actin antibody (25 mM phosphate buffer, pH 7.4).

*Preparation of PZ Polymers and PZ/protein Complexes.* The polymers used in this study were (a) PZs containing 70% (mol) carboxylic acid and 30% (mol) pyrrolidone side groups, i.e., poly[(carboxylatoethylphenoxy)(3-(2-oxo-1-pyrrolidinyl)propylamino)phosphazene], herein called PZ-PYR, or (b) PZ containing 84% (mol) carboxylic acid and 16% (mol) graft 5 kDa polyethylene glycol (PEG) side groups, i.e., poly[di(carboxylatoethylphenoxy)phosphazene]-graft-poly(ethylene glycol), herein called PZ-PEG (Figure 1A). They were synthesized via macromolecular substitution route as described previously [28,29,34].

PZ-PYR or PZ-PEG solutions were then vortexed for 2 min and mixed at 0.6 mg/mL polymer and 0.3 mg/mL protein cargos, including FITC-labeled avidin as a model protein, anti F-actin antibody, or Bax-BH3 peptide as active cargos. The complexes were vortexed for 2 min, complete cell medium was added to reach a concentration of 0.2 mg/mL polymer and 0.1 mg/mL protein, then suspensions were vortexed again for 2 min and used for studies.

*Characterization of PZ/protein Complexes.* Asymmetric Flow Field Flow Fractionation (AF4) characterization was conducted using Postnova AF2000 MT series instrument (Postnova Analytics, Landsberg, Germany) equipped with UV-Vis detector (SPD-20A/20AV, Shimadzu Scientific Instruments, Columbia, MD, USA) and regenerated cellulose membrane (10 kDa molecular weight cutoff, Postnova Analytics, Landsberg, Germany). 25 mM phosphate buffer, pH 7.4 was employed as an eluent. The collected data was processed using AF2000 software (Postnova Analytics, Landsberg, Germany). This technique allows separation of analytes by their size through applying perpendicular flow of mobile phase against the semi-permeable membrane in the analytical cartridge [39]. Although somewhat similar to size exclusion chromatography, AF4 allows characterization of analytes of up to microns in size and minimizes non-specific interactions with a stationary state [39].

*Dynamic Light Scattering*. DLS analysis was conducted using Malvern Zetasizer Nano series, ZEN3600 instrument and Malvern Zetasizer 7.10 software (Malvern Instruments Ltd., Worcestershire, UK). Samples were prepared in 25 mM phosphate buffer, pH 7.4.

*Binding*. Cells grown on coverslips in 24-well plates were incubated with control FITC-avidin, PZ-PYR/FITC-avidin or PZ-PEG/FITC-avidin (0.1 mg/mL FITC-avidin) at 37 °C in complete medium for 0.5, 2 or 5 h. Cells were then washed to remove unbound materials, fixed with 2% paraformaldehyde, nuclei stained with DAPI, and samples mounted on slides with Mowiol and imaged using an Olympus IX81 microscope (Olympus, Inc., Center Valley, PA, USA), 60× oil immersion objective (UPlanApo, Olympus, Inc., Center Valley, PA, USA), ORCA-ER camera (Hamamatsu Corporation, Bridgewater, NJ), and SlideBook™ 4.2 software (Intelligent Imaging Innovations, Denver, CO, USA). Fluorescence images were taken under the green and blue channels to monitor FITC and DAPI respectively, and bright field images provided visualization of cells and cell-cell borders. The FITC-avidin cargo associated with cells was quantified as mean and sum intensity using respective grayscale images and corrected for background intensity, using Image-Pro^®^ v6.3 (Media Cybernetics, Bethesda, MD, USA).

*Uptake*. Cells were incubated with control FITC-avidin, PZ-PYR/FITC-avidin or PZ-PEG/FITC-avidin (green; 0.1 mg/mL FITC-avidin) for the indicated times. To elucidate the uptake mechanism, experiments were conducted at 4 °C vs. 37 °C, in control medium or in the presence of endocytosis inhibitors, including 1 μg/mL filipin to inhibit caveoli, 50 μM monodansylcadaverine (MDC) to inhibit clathrin-dependent endocytosis, or 0.5 μM wortmannin to inhibit macropinocytosis. Additionally, experiments were conducted in the presence of 0.1 mg/mL non-fluorescent avidin or 0.2 mg/mL PZ polymer to block potential binding sites on cells. Both inhibitors and blockers were applied to cells 30 min prior to polymer/protein complexes and then kept during the polymer/protein incubation. Cells were then washed to remove unbound materials, fixed with 2% paraformaldehyde and incubated with mouse IgG conjugated to both biotin and Texas Red. This provided co-staining of green avidin located at the cell surface in red (green + red = yellow), while internalized avidin would not be accessible to this IgG and appeared green. Cell nuclei were stained in blue with DAPI. Samples were mounted onto slides and imaged by fluorescence microscopy as described above. The total number of vesicles per cell, internalized vesicles, and percentage of internalization over total vesicles counted were quantified using a custom macro in Image Pro^®^ (Media Cybernetics, Bethesda, MD, USA) described elsewhere [40].

*Subcellular Localization*. HUVECs were incubated with PZ-PYR/FITC-avidin or PZ-PEG/FITC-avidin (0.2 mg/mL polymer, 0.1 mg/mL protein) for 1 h followed by fixation (1 h trafficking) or then washed to remove non-bound materials and incubated for additional 4 h in complex-free medium to follow the trafficking of pre-bound materials (1 h pulse + 4 h chase = 5 h trafficking). Cells were then fixed, washed, permeabilized, and incubated with anti-Early Endosome Antigen-1 (EEA-1) antibody for endosome colocalization, followed by Texas red-secondary antibody and DAPI. To stain lysosomes, HUVECs were first incubated with 1 mg/mL 10 kDa Texas-red dextran for 45 min, followed by incubation for another 45 min in dye-free medium, then incubation with polymer/protein complexes as described for endosomes, fixation, and fluorescence microscopy imaging. By this technique, the green FITC-avidin colocalizing with red endosomes or lysosomes would appear yellow, which was quantified using a custom macro in Image-Pro 6.3, as described [41,42].

*Functional Protein Delivery*. First, cytosolic delivery of a cell-impermeable antibody was studied. For this, HUVECs were left untreated or incubated at 37 °C with 0.1 mg/mL anti-F-actin alone or complexed with PZ-PYR (0.2 mg/mL polymer) for 1 h pulse, followed by 4 h chase as described above. Then, cells were washed, fixed, permeabilized, and incubated with Texas red-secondary antibody to visualize anti-F-actin. As a positive control, cells were fixed and permeabilized prior to incubation with anti-F-actin, followed by Texas red-secondary antibody. Fluorescence images were taken under the red channel at 60× magnification to image whether anti-F-actin would have bound and thus would have revealed filamentous actin in the cytosol. To determine the mechanism, similar experiments were conducted where HUVECs were pre-treated for 30 min with 300 μM chloroquine (to alkalinize endo-lysosomes) or 0.1 μM latrunculin (to inhibit polymerization of actin into filaments), then incubated with PZ-PYR/anti-F-actin in the presence of these agents.

Second, intracellular delivery of a pro-apoptotic Bax-BH3 peptide was investigated. HUVECs were left untreated or incubated at 37 °C for 1 h pulse with 0.2 mg/mL PZ-PYR alone, 0.1 mg/mL Bax-BH3 peptide alone, PZ-PYR/BH3 peptide (0.2 mg/mL polymer, 0.1 mg/mL protein), or 5 mM hydrogen peroxide (H_2_O_2_) known to damage cells, followed by 4 h chase as described above. Cells were then washed, incubated with LIVE/DEAD assay components containing 0.1 μM calcein AM and 1 μM ethidium homodimer for 30 min. Live fluorescence images were taken under the green and red channels at 20× magnification to monitor live (calcein positive; green) and dead (ethidium positive; red) cells, respectively. They were quantified using Image-Pro 6.3 to calculate the percentage of live (viable) cells from the total cells counted.

*Polymer Cytotoxicity*. HUVECs were left untreated or incubated for 1 h at 37 °C with 0.2 mg/mL PZ-PYR or 5 mM H_2_O_2_ control, followed by a 4 h chase as described above. Cells were then washed, incubated with LIVE/DEAD assay components, and the percentage of live (viable) cells and the number of cells were determined as indicated above.

Furthermore, the integrity of the cell membrane was assessed using the Pierce LDH Cytotoxicity Assay Kit (Thermo Scientific, Asheville, MA, USA). HUVECs grown on 96-well plates were left untreated or incubated for 1 h at 37 °C with 0.2 mg/mL PZ-PYR or 0.1% Triton (known to permeabilize cell membranes), followed by 4 h chase as above. Then, the release of intracellular LDH to the cell medium was determined after separation by centrifugation and by measuring LDH catalytic activity for 30 min per manufacturer’s protocol. Absorbance at 490 nm was measured using SpectraMax M2 Plate Reader and analyzed with SoftMax^®^ Pro Software (both from Molecular Devices, San Jose, CA, USA).

Finally, apoptosis was examined using the Caspase 3/7 Glo assay (Promega, Madison, WI, USA). For this, HUVECs grown on 96-well plates were left untreated or incubated for 1 h with 0.2 mg/mL PZ-PYR or 1 μM staurosporine (known to induce apoptosis). Then, caspase 3/7 revealing reagents were added to cells and measured by luminescence using SpectraMax M2 Plate Reader (Molecular Devices, San Jose, CA, USA) following the manufacturer’s protocol.

*Statistics*. For microscopy, two independent experiments, each one with two independent replicates (total of *n* = 4 wells/condition) were analyzed cell-by-cell, for a total of *n* ≥ 100 cells per condition, randomly selected throughout the whole slide area. For cytotoxicity tests, two independent experiments with 4 replicates/each were conducted. Data were calculated as mean ± standard error of the mean (SEM). Statistical significance for two-way comparisons was determined using Student’s *t*-test, *p* < 0.05.

## 3. Results

### 3.1. Assembly of Supramolecular Protein-Loaded PZ Constructs

First, molecular interactions of PZ-PYR and PZ-PEG polymers (Figure 1A) with proteins were investigated as the formation of supramolecular complexes between macromolecular carrier and protein cargo constitutes an important pre-requisite for successful intracellular delivery. FITC-avidin, a 68 kDa protein, was chosen as a model cargo since this fluorescent tag would allow us to easily trace delivery of the protein within cells. Polymer/protein formulations were prepared in aqueous solutions at neutral pH by simple mixing of the components and were then analyzed using asymmetric flow field flow fractionation (AF4) method. Figure 1B displays AF4 profiles for FITC-avidin, PZ-PYR carrier, and the resulting PZ-PYR/FITC-avidin formulations. As expected, PZ-PYR was not detected at a 495 nm wavelength (flat green line), but its 210 nm profile shown as a dashed gray trace revealed a broad peak with a maximum at 8 min elution time. FITC-avidin displayed a narrow peak at 7 min (red trace, Figure 1B), but its formulation with PZ-PYR (“invisible” at 495 nm) revealed a broad peak (brown trace), which resembled the polymer peak (gray trace), but with a significantly longer elution time of 11 min. This “visualization” of PZ-PYR by fluorescently labeled protein demonstrates its non-covalent assembly with the polymer carrier, which was also confirmed by the disappearance of the “unbound” avidin peak at 7 min. The location of the peak (11 min vs. 7 min for avidin and 8 min for PZ-PYR peak) suggests a significant increase in the size of supramolecular assembly compared to polymer or protein components (longer elution time in AF4 indicates larger sizes as smaller molecules tend to move faster in the analytical cartridge).

This increase in size is also evident from DLS data (Figure 1C), which shows the peak average values for the inter-molecular assembly around 200 nm, as opposed to 35 nm for PZ-PYR and 6 nm for avidin. In addition, both, PZ-PEG and PZ-PYR display strong avidity to FITC-avidin, as calculated by the decrease of protein peaks on the fractogram. No decrease was observed (100% binding efficiency) which results in a complete binding of this protein to the polymer carrier (Figure 1D).

AF4 analysis was also applied to a 150 kDa anti-F-actin antibody and a 2.2 kDa Bax-BH3 peptide employed later in this study, and the corresponding results are described in Section 3.6.

### 3.2. Binding of PZ/Protein Complexes to Different Cell Types

**Figure 2:**
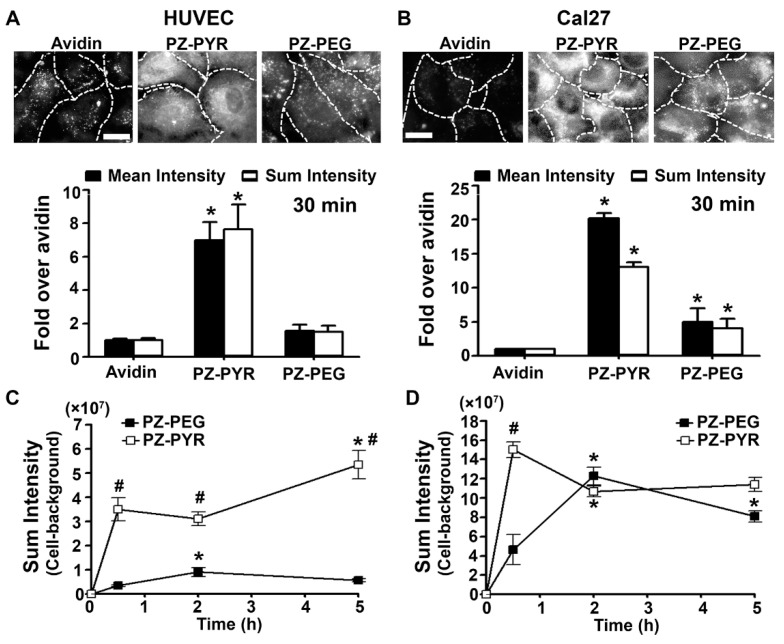
Binding of PZ/protein complexes to different cell types. (**A**) Human Umbilical Vein Endothelial Cells (HUVECs) or (**B**) Cal27 cells, incubated for 30 min at 37 °C with FITC-avidin alone or complexed to PZ-PYR or PZ-PEG. Fluorescence microcopy images (top) and image quantification of the mean intensity and sum intensity (bottom) are shown. Dashed lines = cell borders as seen by bright field. Scale bar = 10 µm. Data are mean ± SEM. * *p* < 0.05 relative to control avidin. (**C**,**D**) Similar experiments comparing different incubation times in (**C**) HUVECs or (**D**) Cal27 cells, quantified as fluorescence sum intensity. Data are mean ± SEM. # *p* < 0.05 relative to PZ-PEG, * *p* < 0.05 relative to the previous time point.

We selected two different human cell types for this study: HUVECs, primary cells of endothelial origin that represent a control condition, and Cal27, an established oral adenocarcinoma cell line. In endothelial cells, complexation with PZ-PYR enhanced by 7-fold the cell association of FITC-avidin by as early as 30 min after the start of the incubation period, measured as both fluorescence mean intensity or sum intensity (Figure 2A). In Cal27 cells, treatment with PZ-PYR/FITC-avidin increased cell association even further, from 15- to 25-fold depending on the parameter measured (Figure 2B). For both cell types, PZ-PEG/FITC-avidin associated to a lesser extent than the PZ-PYR/FITC-avidin to cells, e.g., 4- to 5-fold less in HUVECs and 3- to 4-fold less in Cal27 cells.

Incubation of PZ/protein complexes with cells for additional time periods revealed a different kinetics and maximal binding for both polymers and cell types. In both cell types, cell association of PZ-PYR/FITC-avidin was faster than PZ-PEG/FITC-avidin (compare the 0-to-30 min slopes in Figure 2C,D). However, after that time point, PZ-PEG/FITC-avidin continued accumulating in Cal27, reaching a level similar to PZ-PYR/FITC-avidin by 2 h, while in HUVECs, PZ-PEG/FITC-avidin barely accumulate any further by 2 h or 5 h.

### 3.3. Uptake of PZ/Protein Complexes in Different Cell Types

**Figure 3:**
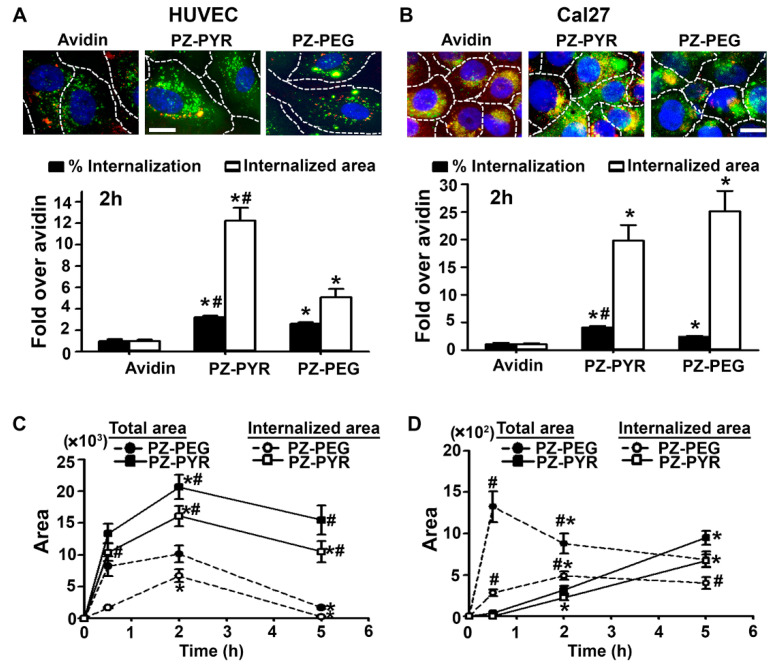
Uptake of PZ/protein complexes in different cell types. (**A**) HUVECs or (**B**) Cal27 cells incubated for 30 min at 37 °C with green FITC-avidin alone or complexed to PZ-PYR or PZ-PEG. Cells were washed, fixed, and incubated with Texas Red-labeled biotin-IgG to co-stain avidin on the cell surface in red (green + red = yellow) vs. internalized avidin (green alone). Blue = cell nuclei stained with DAPI. Dashed lines = cell borders as seen by bright field. Scale bar = 10 µm. Data are mean ± SEM. * *p* < 0.05 relative to avidin. (**C**,**D**) Similar experiments comparing different incubation times in (**C**) HUVECs or (**D**) Cal27 cells. Data are mean ± SEM. ^#^ *p* < 0.05 comparing both polymers, * *p* < 0.05 relative to the previous time point.

We then focused on the ability of PZ/protein complexes to enter cells (Figure 3), for which we used a double labeling method that allows us to distinguish surface bound (green + red = yellow) vs. internalized (green alone) avidin fractions (see Section 2). The percentage of internalized FITC-avidin was similarly enhanced (3- to 4-fold) by complexation to PZ-PYR and PZ-PEG in both cell types (Figure 3A,B). Hence, no major differences in the uptake capacity of cells was observed for these polymers. However, according to the total binding observed before when total internalized FITC-avidin was examined, higher levels were observed for Cal27 cells over HUVECs, i.e. 20-fold and 12-fold for PZ-PYR/FITC-avidin over control FITC-avidin, respectively (Figure 3A,B). Additionally, PZ-PEG was 2.4-fold less efficient than PZ-PYR regarding internalization of FITC-avidin in HUVECs, while this was not the case for Cal27 cells (1.3-fold higher compared to PZ-PYR; Figure 3A,B).

Examination of uptake over different time points revealed that in HUVECs, the rate of uptake of the PZ-PYR/FITC-avidin was faster than PZ-PEG/FITC-avidin (compare 0-to-30 min slopes in Figure 3C). The maximal uptake for both PZ-PYR/FITC-avidin and PZ-PEG/FITC-avidin formulations was seen at 2 h (Figure 3C), while maximal binding was already observed at 30 min (Figure 2C), expected as uptake is subsequent to binding. In addition, a decrease was found from 2 h to 5 h for both polymers, but more acute for PZ-PEG (compare the slopes in Figure 3C). In Cal27 cells, the rate of uptake of the PZ-PEG/FITC-avidin was faster than PZ-PYR/FITC-avidin (compare 0-to-30 min slopes in Figure 3D), but the uptake of PZ-PYR/FITC-avidin continued to increase over time while that of PZ-PEG/FITC-avidin remained constant after 2 h (Figure 3D).

### 3.4. Mechanism of Uptake of PZ/Protein Complexes

**Figure 4:**
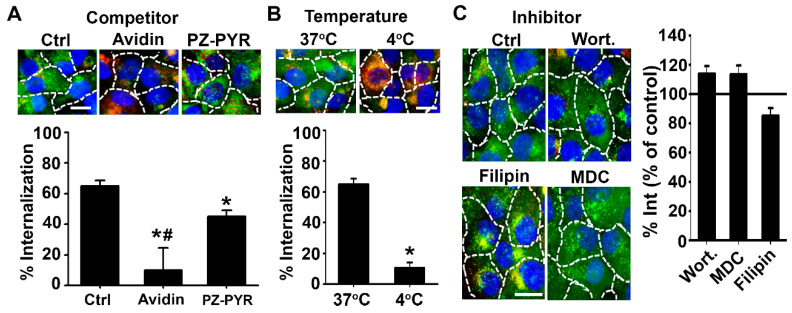
Mechanism of uptake of PZ/protein complexes by cells. Cal27 cells incubated for 2 h with FITC-avidin complexed to PZ-PYR: (**A**) in control (Ctrl) cell medium vs. medium containing avidin or polymer (competitors), (**B**) at 4 °C vs. 37 °C, or (**C**) in control cell medium vs. medium containing inhibitors of macropinocytosis (wortmannin, Wort), caveolar pathway (filipin) or the clathrin pathway (MDC). All cells were then fixed after incubation, surface-bound FITC-avidin was co-stained with a Texas Red biotin-IgG (green + red = yellow), and nuclei were stained with blue DAPI. Dashed lines = cell borders as seen by bright field. Scale bar = 10 µm. Fluorescent images and their quantification are shown. Data expressed as mean ± SEM. * *p* < 0.05 relative to the respective control condition. # *p* < 0.05 relative to PZ-PYR.

Then, the mechanism of uptake of these complexes into cells was studied as described above. First, cells were pre-incubated with excess quantities of either non-fluorescent avidin or polymer (PZ-PYR) alone to compete for any possible interaction sites with cells, and then polymer/FITC-avidin complex was added to the cell medium to compare uptake vs. cells that did not receive blockers (Figure 4). Surprisingly, both cargo (avidin) as well as the polymer (PZ-PYR) decreased uptake of PZ-PYR/FITC-avidin by 63% and 30%, respectively, compared to the non-blocked control (Figure 4A).

Internalization of PZ-PYR/FITC-avidin by cells was inhibited by 83% at 4 °C compared to 37 °C (Figure 4B), indicating that uptake occurs through an active mechanism. However, incubation of the PZ-PYR/FITC-avidin complex with cells in the presence of individual inhibitors of endocytic pathways, such as monodansylcadaverine (MDC) which affects clathrin-mediated endocytosis, filipin that affects the caveolar-mediated pathway or wortmannin (Wort) that inhibits macropinocytosis, did not cause a decrease in uptake compared to the control condition (Figure 4C). This phenomenon is typically observed when uptake is not specific to one pathway but rather any pathway used by the cell can lead to uptake at that point in time.

### 3.5. Endo-Lysosomal Trafficking of Internalized PZ/Protein Complexes

**Figure 5:**
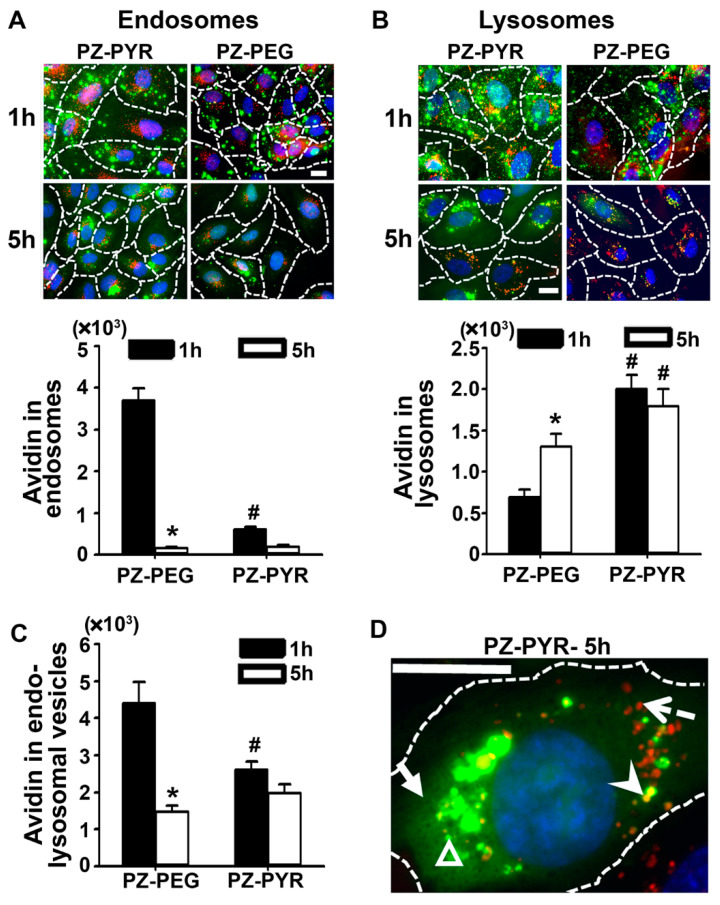
Subcellular trafficking of PZ/avidin complexes. HUVECs were incubated for 1 h at 37 °C with FITC-avidin complexed to PZ-PYR or PZ-PEG and then processed for fluorescence microscopy or incubated for additional 4 h in complex-free cell medium (total 1 + 4 = 5 h) followed by sample processing. (**A**) Cells were permeabilized and early endosomes immunostained to appear red or (**B**) lysosomes were labeled by pre-incubating cells with 10 kDa Texas Red dextran prior to incubations with polymer/FITC-avidin complex. In both cases, colocalization of FITC-avidin with these compartments appears yellow (green + red) and is expressed as the area of pixels colocalizing with each compartment. (**C**) Total FITC-avidin in vesicular (endosomal + lysosomal) compartments, expressed as total area of colocalizing pixels. (**D**) High-magnification image of FITC-avidin delivered by PZ-PYR at 5 h, showing lysosomes which do not contain FITC-avidin (red; dotted arrow), FITC-avidin in lysosomes (yellow; arrowhead); FITC-avidin in dot-like vesicular compartments that are not lysosomes (green dots; open arrowhead), and FITC-avidin in the cytosol (solid arrow). All data are expressed as mean ± SEM. * *p* < 0.05 relative to 1 h. # *p* < 0.05 relative to PZ-PEG. Dashed lines = cell borders as seen by bright field. Scale bar = 10 µm.

The subcellular fate of complexes was then studied using a pulse-chase protocol, wherein cells were treated with the polymer/FITC-avidin complex for 1 h to allow cell association (pulse), followed by replacing the complex-containing cell medium with complex-free medium to enable trafficking of pre-associated materials for an additional 4 h (chase; total pulse + chase time = 5 h) (Figure 5). Subsequent immunostaining of early endosomes in red enabled visualization of colocalization of green FITC-avidin in these compartments as yellow (Figure 5A). In the case of PZ-PEG/FITC-avidin, endosomal colocalization was observed at 1 h, then it decreased by 95% at 5 h. For PZ-PYR/FITC-avidin, much lower endosomal colocalization was seen at 1 h compared to PZ-PEG, which was not significantly decreased at 5 h.

As per colocalization with lysosomes, this was then studied by pre-incubation of cells with a 10 kDa Texas-Red dextran, which is known to traffic to and remain in lysosomes in these cells [41,42]. Interestingly, PZ-PYR/FITC-avidin co-localized to a higher extent with lysosomes compared to PZ-PEG/FITC-avidin at 1 h (Figure 5B). However, while the colocalization of PZ-PEG/FITC-avidin with lysosomes increased from 1 h to 5 h, it remained unchanged for PZ-PYR/FITC-avidin (Figure 5B). Since endosomes are a preceding compartment for internalized materials in transit to lysosomes, this data suggests that the endo-lysosomal trafficking of PZ-PYR/FITC-avidin within cells may be faster than that of PZ-PEG and/or that this complex escapes this route. Quantification of FITC-avidin present in both endosomal+lysosomal vesicles at 1 h yielded significantly lower endo-lysosomal colocalization for PZ-PYR/FITC-avidin compared to PZ-PEG/FITC-avidin (Figure 5C), although its total cell binding and uptake had been higher (Figure 2A and Figure 3A). Hence, a significant fraction of the internalized PZ-PYR/FITC-avidin must be in another compartment, which seemed to be the cytosol, as visualized in high-magnification microscopy images (solid arrow compared to arrowhead in Figure 5D). The total amount of FITC-avidin contained in vesicles (endosomes+lysosomes) decreased by 3-fold from 1 h to 5 h when delivered by PZ-PEG, while it only decreased by 1.3-fold when delivered by PZ-PYR, suggesting that the latter polymer may expose FITC-avidin to lower degradation within cells, in agreement with endo-lysosomal escape.

### 3.6. Cytosolic Delivery of Active Cargos by PZ Polymers

To verify the endo-lysosomal escape of cargo into the cytosol of cells, an antibody against filamentous actin (anti F-actin) was chosen because it is cell-impermeable, as most other antibodies, and it represents a large protein cargo. First the avidity of anti-F-actin to PZ polymers was studied by AF4 analysis and calculated by decrease of peptide peaks on the fractogram (Figure 1D). Anti-F-actin displayed a strong avidity to PZ-PYR, resulting in complete complexation of protein to polymer. Complexation to PZ-PEG polymer was less efficient (30% of all protein), yet still possible (Figure 1D). Since PZ-PYR was the polymer that seemed to exhibit cytosolic release and was more efficient at forming complexes with this antibody, PZ-PYR was chosen for this study.

**Figure 6:**
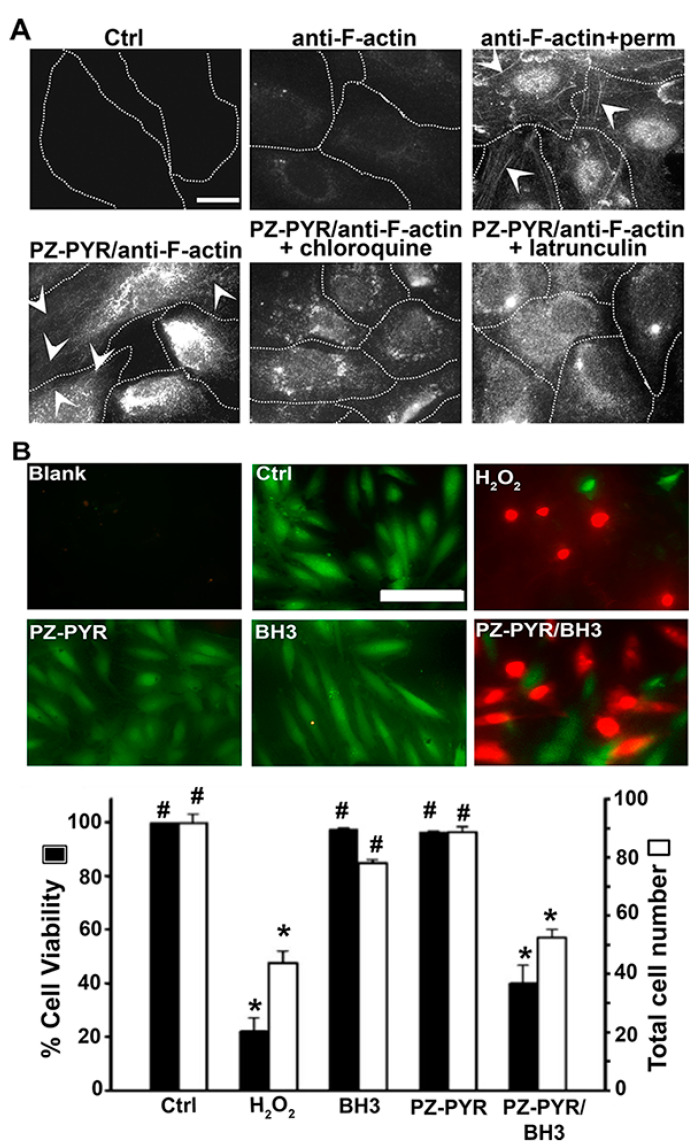
Cytosolic delivery of active proteins by PZ polymer. (**A**) HUVECs were incubated for 1 h at 37 °C with control medium (Ctrl), anti-F-actin antibody alone or PZ-PYR/anti-F-actin, in the absence or presence of chloroquine (to inhibit endo-lysosomal acidification) or latrunculin (to inhibit polymerization of F-actin), followed by content removal and 4 h incubation with fresh cell medium. Post permeabilization of cells, F-actin was stained using a fluorescent secondary antibody and visualized by fluorescence microscopy. Anti-F-actin + perm. is a control where anti-F-actin was incubated with cells previously permeabilized. Arrowheads = actin fibers. Scale bar = 10 µm. (**B**) HUVECs were incubated for 1 h at 37 °C with control medium (Ctrl), H_2_O_2_ to induce cell death, PZ-PYR polymer alone, and Bax-BH3 peptide alone or complexed to PZ-PYR, followed by content removal and 4 h incubation with fresh cell medium. Staining with the live-dead viability reagent was conducted to stain live cells in green and dead cells in red, for fluorescence microscopy visualization. Scale bar = 50 μm. Total number of cells per image (white bars) and % viability expressed as the percentage of live cells among total cells (black bars). Data are mean ± SEM. * *p* < 0.05 relative to the untreated control, # *p* < 0.05 relative to H_2_O_2_.

As expected, incubation of cells with anti F-actin alone did not show characteristic intracellular staining of filamentous actin, while permeabilization of cells prior to antibody incubation demonstrates this pattern (Figure 6A). When anti-F-actin was delivered by PZ-PYR complexes, filamentous actin could be visualized, albeit at a lower extent than permeabilizing cells prior to treatment (Figure 6A). In addition, chloroquine, a mild base that prevents endo-lysosomal acidification, prior to treatment with PZ-PYR/anti-F-actin abolished the visualization of typical filamentous actin pattern (Figure 6A). This further confirms that endo-lysosomal acidification was necessary for PZ-PYR/anti-F-actin to escape into the cytosol. Finally, latrunculin, an inhibitor of filamentous actin, also abolished the visualization of actin fibers, confirming the selectivity o anti-F-actin for this antigen (Figure 6A). Taken together, these data demonstrate that large protein cargo complexed to PZ-PYR can be intracellularly delivered, escape into the cytosol, and retain functionality (binding to its antigen, in this case).

To further verify whether other protein cargoes can be delivered to the cell cytosol by PZ-PYR, the pro-apoptotic Bax-BH3 peptide was chosen. The molecular targets of Bax-BH3 are Bcl-2 and Bcl-X_L_, anti-apoptotic proteins present on the outer membrane of mitochondria, facing the cytosol, whereby binding of this peptide to its targets is known to induce apoptosis leading to cell death [43,44]. Thus, cytosolic delivery of the Bax-BH3 peptide should enable this function. However, this 20-amino acid peptide is impermeable to cells, for which its effective delivery requires direct injection into this compartment [43,44,45]. Hence, this example can also serve to verify whether active cargo can be delivered to the cytosol by PZ polymers. The avidity of Bax-BH3 peptide to PZ-PYR was similarly examined by AF4 analysis. Linear PZ-PYR showed only minimal decrease in case of Bax-BH3 (80% of protein molecules remained bound; Figure 1D). Graft PZ-PEG copolymer was less efficient, but still approximately 20% of Bax-BH3 were still bound to the polymer. Once again, since PZ-PYR was the polymer that seemed to exhibit cytosolic release and it bound a higher Bax-BH3 peptide content, PZ-PYR/Bax-BH3 complex was chosen for this study.

Cells were incubated with this complex or respective controls, followed by a live-dead viability assay to examine cell death induced by effective Bax-BH3 delivery. Incubation of cells with the Bax-BH3 peptide alone or the PZ-PYR polymer alone did not decrease the viability or number of cells compared to the untreated control, as expected (Figure 6B). However, incorporation of the peptide in the PZ-PYR complex reduced the cell viability by 60% and the total cell number by 40%, which is comparable to the effect of H_2_O_2_ control. This indicates that the peptide cargo reaches the cytosol in an active form, where it elicits an apoptotic effect, likely by interacting with proteins on the outer (cytosolic side) mitochondrial membrane.

### 3.7. Cytotoxicity of PZ Polymers

**Figure 7:**
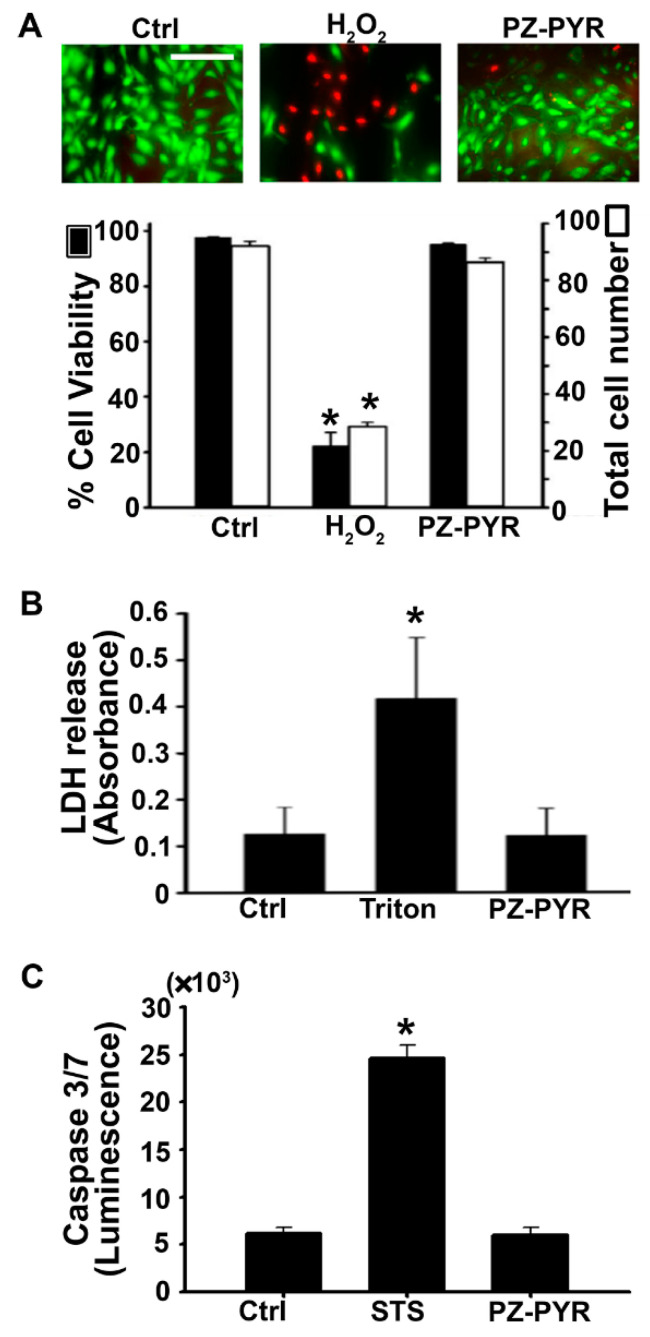
Cytotoxicity of polyphosphazene polymers. (**A**) HUVECs were treated with regular medium (Ctrl), 5 mM hydrogen peroxide (H_2_O_2_) to induce cell death, or PZ-PYR for 1 h following which the treatment was removed, and a live-dead viability assay was conducted where live cells appear green whereas dead cells appear red in fluorescent images. Data indicates the mean ± SEM total number of cells per image (white bars) as well as the % viability expressed as the percentage of live cells among the total cells (black bars). Scale bar = 50 μm. (**B**) HUVECs were incubated with regular medium (Ctrl), 0.1% triton to permeabilize cell membranes, or PZ-PYR for 1h following which the medium was removed and subjected to measurement of lactate dehydrogenase released into the medium. Data expressed as mean ± SEM of absorbance at 490 nm. (**C**) HUVECs were incubated with regular medium (Ctrl), 1 μM staurosporine (STS) to induce apoptosis, or PZ-PYR for 1 h following which the Caspase 3/7 in the cell + medium fraction was determined. Data expressed as mean ± SEM of luminescence. * *p* < 0.05 relative to the untreated control.

Finally, PZ polymer toxicity was determined. First, a Live/Dead viability assay that relies on the metabolic state and membrane permeability of the cells was applied, wherein green fluorescence of calcein within cells can be attributed to intact intracellular esterase activity indicating viable cells. On the other hand, red fluorescent nuclei can be attributed to damaged cell membranes which permit staining of nuclei by the otherwise cell impermeable ethidium homodimer. As expected, treatment with H_2_O_2_ significantly reduced (66–80% decrease) both the cell viability and number of cells (Figure 7A). In contrast, the PZ-PYR did not change these parameters (96% of control values). In agreement with this result, cells incubated with PZ-PYR did not release intracellular LDH to the extracellular cell medium (97% of control values; Figure 7B), while cells incubated with the non-ionic surfactant Triton X-100 did (3.3-fold increase), verifying that the membranes of cells remained intact upon incubation with PZ-PYR.

Finally, the potential of PZ-PYR to induce apoptosis in cells as an early event prior to loss of cell viability was also determined. This assay was based on the measurement of effector caspases 3 and 7, definitive markers of apoptosis induction in cells. If caspases are generated within cells following an apoptotic stimulus, the assay measures luminescence due to cleavage of a pro-luminescent substrate by these caspases. As expected, staurosporine, an apoptosis inducer, caused a 4-fold increase in luminescence compared to the untreated control, while PZ-PYR did not (97% of control value; Figure 7C). Taken together, these data indicate that PZ polymers used in this study were not cytotoxic to cells at concentrations where delivery and subsequent effect of functional proteins was observed.

## 4. Discussion

Delivery of protein therapeutics to intracellular sites remains a major challenge. Strategies that take advantage of nanomaterials offer an attractive alternative towards this goal, but many of these systems suffer from toxicity, restrictions of cargo size and type, or involve modification of the protein cargo resulting in detrimental structural and/or activity changes; thus, a platform-technology that can be broadly applicable to different cargos remains elusive [3,5,7,17]. As a result, only a handful of protein therapeutics whose targets are intracellular have been found to be effective [24,25]. Most clinical trials being conducted relate to custom-designed, covalently-conjugated, protein-cargo systems based on CPPs, which have been researched for over 30 years and represent the most commonly studied intracellular delivery system [10]. Focusing on this challenge, in this study we have investigated PZ polymers since they are biodegradable [31,32], can bind a variety of proteins through non-covalent interactions [29,34], and can be further functionalized to tune parameters such as extending in vivo half-life using PEG [32].

As demonstrated here, both PZ-PYR and PZ-PEG facilitated interaction of protein cargo, i.e., FITC-avidin used as a model protein, with cells (Figure 2). This was expected based on the characteristics of these polymers. Since PZ polymers interact with proteins and other molecules (e.g., lipids, polysaccharides, etc.) via non-covalent electrostatic and hydrogen bonding [27,28,34], it is expected that these polymers would interact with such elements on the cell surface, facilitating protein delivery. Interestingly, in studies to determine the mechanism of interaction with cells, it was observed that both avidin and polymer pre-incubated with cells and present during the treatment with polymer/protein complexes decreased uptake of the complex by cells, with avidin exerting a more acute influence (Figure 4A). This can be explained based on the loading capacity of avidin in the polymer and the fact that this interaction must reach a dynamic equilibrium. When non-fluorescent avidin was used as a competitor, it possibly displaced or exchanged with FITC-avidin from the complex. Since polymer/non-fluorescent avidin would not be visible by fluorescence microscopy and freed FITC-avidin would not efficiently interact with cells, this would explain the observed result. When PZ-PYR was used as a competitor, FITC-avidin from the PZ-PYR/FITC-avidin complex may also exchange into the competitor polymer, so that the same total amount of FITC-avidin is loaded in a greater amount of polymer and this “loading dilution” effect would not affect cell interaction but lower the amount of cargo that was delivered. Alternatively or simultaneously, protein-free PZ-PYR may be interacting with the cell surface similarly as PZ-PYR/FITC-avidin does, blocking and/or competing off binding of PZ-PYR/FITC-avidin.

Importantly, data revealed that these polymer/protein complexes facilitated the rapid interaction and internalization of FITC-avidin in cells, although the efficiency of these processes depended on the particular polymer and cell type examined (Figure 2 and Figure 3). In general, PZ-PEG was slower and reached lower levels than PZ-PYR for these processes. This finding is not surprising since PEG chains on carriers are known to interfere with cell surface interactions, possibly owing to the formation of a hydration layer and steric hindrances [46]. However, interestingly, slower and lower binding/uptake of PZ-PEG/FITC-avidin was much more noticeable in control endothelial cells than adenocarcinoma cells. This result can be speculatively explained by the mechanism followed by PZ polymers to enter cells (Figure 4B,C). This was governed by an active process (Figure 4B), but no specific pathway was found to be involved, since none of the endocytosis inhibitors used hindered uptake by cells. This means these complexes do not actively induce endocytosis, but rather piggyback into the cell by any endocytic route the cell is using at the moment. Since PZ polymers could interact with elements of the cell membrane through the non-covalent interactions described above, then as a cell performs endocytosis to uptake molecules from the cell medium or recycle its membrane components, PZ/protein complexes would simply enter the cell attached to the membrane at sites where endocytic vesicles are being formed. As such, blocking clathrin, caveolar, or macropinocytic pathways individually would not necessarily block polymer/protein uptake since they could piggyback along the pathways that are left uninhibited, in a compensatory-like manner. This is in contrast to polymers that bear functional groups such as guanidium and boronate on cationic backbones to act as protein cargo “glue” and increase stability of the complexes [2]. For instance, guanidyl-modified polyethyleneimine polymers have also been shown to be effective for intracellular protein delivery through interaction with membrane phospholipids, but do not utilize compensatory mechanisms in case of pathway inhibition [24]. Coming back to the question on why PZ-PEG/FITC-avidin was slower and reached lower levels in endothelial cells compared to adenocarcinoma cells, this could be explained if the former cell type had a lower endocytic activity compared to the latter cell type. In fact, enhanced endocytosis is a feature recognized in many cancer types [47]. This phenomenon applies to all drug delivery systems, including CPPs [12], because different cell types exhibit different sizes/surface areas and different shapes/morphology, as well as a different endocytic activity and/or use different endocytic pathways at different extents depending on their biological function, the signals they receive from the environment, their metabolic rate, and/or their pathophysiological states [47,48]. This is true even for drug delivery systems targeted to specific cell-surface receptors, e.g., the same ICAM-1 targeted polymer nanoparticles have been shown to traffic mainly to lysosomes in neuronal-like cells [49], fibroblasts [50], or endothelial cells grown without a basolateral free-surface [50], while they mainly traffic across the cell body via transcytosis in endothelial cells or epithelial cells grown with a basolateral free-surface [51,52]. Similarly, endocytic pathways can be altered, either decreased or increased, depending on the pathophysiological state of cells [48], e.g., uptake via clathrin or caveolar pathways, but not the CAM pathway, was decreased in fibroblasts affected by various genetic diseases called lysosomal storage disorders [41]. Therefore, based on this differential cellular interaction/internalization efficacy of PZ-PYR and PZ-PEG, each of these polymers could offer different advantages depending on the cell target intended for a particular therapeutic application.

Following entry into cells by endocytosis, protein delivery systems are often retained in the endo-lysosomal compartment and cannot escape into the cytosol to traffic to or reach other intracellular targets [2,5,8,24,45]. With regard to the subsequent endo-lysosomal trafficking of internalized PZ complexes, we again saw a difference in the kinetics of the process between the two polymers, with the PZ-PYR/FITC-avidin trafficking rapidly to the lysosomal compartment, as evidenced by a low colocalization with endosomes at 1 h or 5 h, while their localization with lysosomes was slightly higher compared to endosomes and sustained over this time (Figure 5A,B). PZ-PEG/FITC-avidin was more significantly found in endosomes by 1 h, then it accumulated in the lysosomes over time. This would be expected from the interaction/uptake kinetics observed for these two polymers. However, interestingly, when observing the internalized fraction of PZ-PEG/FITC-avidin (Figure 3A,C), it seems that all of the internalized cargo was retained in the endo-lysosomal compartment (Figure 5C) at 1 h and 5 h. For PZ-PYR/FITC-avidin, although there was some colocalization with the endo-lysosomal compartment, this was a much smaller fraction compared to what was internalized, e.g. only ~20% of the internalized cargo was present in the endo-lysosomal compartment (Figure 3C vs. Figure 5C), indicating that the remaining fraction escaped this compartment. A closer look at the internalization kinetics revealed that there is ~30% loss of the internalized PZ-PYR/FITC-avidin from 2 to 5 h (opposed to ~96% loss of PZ-PEG/FITC-avidin), indicating that the remaining 50% possibly escaped into the cytosol. The fact that significant reduction of PZ-PEG/FITC-avidin is found over time (Figure 3C) suggests there is time-dependent degradation of this complex, possibly as a consequence of its endo-lysosomal retention. This differential ability of PZ-PYR compared to PZ-PEG to enable endo-lysosomal escape may be speculatively explained considering PEG may be shielding PZ functional groups, preventing the interaction of the polymer with endo-lysosomal membranes, just as observed for interaction with the cell membrane (Figure 2 and Figure 3). The increased punctate-like distribution observed for PZ-PEG/FITC-avidin vs. the more diffuse distribution of PZ-PYR/FITC-avidin (pictures in Figure 2A,B) supports their respective more vesicular vs. more cytoplasmic distribution, respectively.

Consequently, PZ-PYR was examined for its ability to facilitate cytoplasmic delivery of active protein cargos into cells (Figure 6). Data on PZ-PYR-based delivery of a large protein, anti-F-actin, demonstrated release of this antibody into the cytosol and binding to filamentous actin (Figure 6A). Expectedly, said delivery was not as prominent as when the antibody was applied on permeabilized cells, as this represents a scenario where cell membranes do not constitute a barrier for antibody penetration. In fact, this suggests that PZ-PYR did not permeabilize the cell membrane, in agreement with its capacity for cytosolic delivery from endo-lysosomal compartments after internalization. This was verified by the fact that chloroquine, a mild base that prevents endo-lysosomal acidification, hindered cytosolic delivery of anti-F-actin antibody (Figure 6A). Further, data on the Bax-BH3 peptide showed ≈60% reduction in cell viability which would only be possible if the peptide escaped from the endo-lysosomal compartment into the cytosol and then bound its molecular target on the cytosol-facing outer membrane of mitochondria (Figure 6B). Such functional experiments are more reliable and preferable compared to only tracing a labeled cargo using flow cytometry or microscopy [53]. The effect observed was similar to other studies where the peptide was microinjected into cells [43] or introduced by conjugation with cell penetrating peptides [54]. However, most such membrane permeabilizing methods are limiting due to toxicity, while PZ-PYR used here did not alter the cell membrane or viability (Figure 7), as demonstrated by the fact that ethidium homodimer used in the Live/Dead assay did not penetrate into cells, cells maintained their metabolic activity since calcein AM was cleaved demonstrating intracellular esterase activity (Figure 7A), LDH was not released from cells, and the polymer did not induce apoptosis as observed by a lack of induction of effector caspase 3 and 7 (Figure 7B,C).

Hence, PZ/protein complexes represent a platform-based delivery system that is non-toxic, can form complexes with different types of proteins, ranging in molecular weights from 2 kDa to 150 kDa, carry them into cells and deliver them to the endo-lysosomal route (PZ-PEG) or the cytosol (PZ-PYR; Figure 5), maintaining their activity (Figure 6). Other delivery systems that have been investigated for their ability to non-covalently assemble into supramolecular complexes broadly applicable across different protein cargo types are cationic polymers functionalized with guanidium [24], polymeric protein transduction domain mimics (PTDMs) [25], fluoroamphiphilic polymers [26], Pep-1 [13], CPP adaptors [11], which have been found to be efficient for in vitro cytosolic delivery using reporter molecules. Of particular note is that the polymer:cargo ratio for this PZ delivery system ranged from 2:1 to 2.5:1, significantly higher than CPPs that use a 10:1 CPP:cargo ratio [12], and most other systems designed to carry proteins, are not aimed for intracellular delivery [4,27,55]. Therefore, these PZ polymers will add to a very narrow repertoire of polymers that can spontaneously self-assemble with a number of different types of cargo such as peptides, proteins and antibodies, using a simple mixing protocol, and deliver them to different cell types. The in vivo pharmacokinetics of these polymers, the use of targeting moieties to enhance their tissue specificity, and the incorporation of sub-cellular targeting ligands will be explored in future studies.

## 5. Conclusions

PZ-PYR and PZ-PEG polymers can efficiently carry, by non-covalent complexation, significant amounts of large and small protein cargos, demonstrated here using avidin, anti-F-actin antibody and Bax-BH3 peptide. These polymers can also facilitate protein cargo interaction with and uptake into cells via multiple endocytic pathways and provide trafficking through the intracellular endo-lysosomal route. As for most other drug delivery systems, including CPPs, the efficacy of this cell interaction, internalization, and endo-lysosomal trafficking depends on the particular polymer and cell type examined. A linear PZ-PYR was internalized faster and reached higher levels compared to PEGylated graft copolymer PZ-PEG, yet this was more evident in control endothelial cells compared to adenocarcinoma cells. Hence, these polymers may offer different advantages depending on the cell target intended for a particular therapeutic application. Protein cargo carried by PZ-PEG tended to be retained in endo-lysosomal vesicles, which can be used for delivery of recombinant lysosomal enzymes employed for treatment of lysosomal storage diseases [23]. Instead, PZ-PYR offered endo-lysosomal escape without compromising cell viability, enabling cytosolic delivery of active protein cargo. This was demonstrated in this study by cytosolic delivery of F-actin antibody as well as apoptotic activity of Bax-BH3. Both PZ polymers facilitated protein delivery in primary cells and established cell lines of various lineages, shown here using endothelial and epithelial cell types, and different physiological states, demonstrated using control vs. cancer cell examples. Therefore, these anionic biodegradable PZ polymers have the potential to be developed as carriers of protein cargo for intracellular delivery.

## Data Availability

Data available upon request.
